# Assessment of *Plasmodium* antigens and CRP in dried blood spots with multiplex malaria array

**DOI:** 10.1007/s12639-020-01325-2

**Published:** 2021-01-03

**Authors:** Ihn Kyung Jang, Sara Aranda, Rebecca Barney, Andrew Rashid, Muhammad Helwany, John C. Rek, Emmanuel Arinaitwe, Harriet Adrama, Maxwell Murphy, Mallika Imwong, Stephane Proux, Warat Haohankhunnatham, Xavier C. Ding, François Nosten, Bryan Greenhouse, Dionicia Gamboa, Gonzalo J. Domingo

**Affiliations:** 1 Diagnostics Program, PATH, Seattle, WA USA; 2 Infectious Diseases Research Collaboration, Kampala, Uganda; 3 Department of Medicine, University of California at San Francisco, San Francisco, CA USA; 4 Faculty of Tropical Medicine, Department of Molecular Tropical Medicine and Genetics, Mahidol University, Bangkok, Thailand; 5 Faculty of Tropical Medicine, Mahidol-Oxford Tropical Medicine Research Unit, Shoklo Malaria Research Unit, Mahidol University, Mae Sot, Thailand; 6 The Foundation for Innovative New Diagnostics, Geneva, Switzerland; 7 Nuffield Department of Medicine, Centre for Tropical Medicine and Global Health, University of Oxford, Oxford, UK; 8 Departamento de Ciencias Celulares y Moleculares, Facultad de Ciencias y Filosofía, Instituto de Medicina Tropical Alexander von Humboldt, Universidad Peruana Cayetano Heredia, Lima, Peru

**Keywords:** Malaria, HRP2, LDH, Multiplex, Immunoassay, Dried blood spot

## Abstract

Dried blood spots (DBS) typically prepared on filter papers are an ideal sample type for malaria surveillance by offering easy and cost-effective methods in terms of sample collection, storage, and transport. The objective of this study was to evaluate the applicability of DBS with a commercial multiplex malaria assay, developed to concurrently measure *Plasmodium* antigens, histidine-rich protein 2 (HRP2), *Plasmodium* lactate dehydrogenase (pLDH), and a host inflammatory biomarker, C-reactive protein (CRP), in whole blood. The assay conditions were optimized for DBS, and thermal stability for measurement of *Plasmodium* antigens and CRP in dried blood were determined. Performance of the multiplex assay on matched DBS and whole blood pellet samples was also evaluated using the clinical samples. The results indicate the acceptable performance in multiplex antigen detection using DBS samples. At cutoff levels for DBS, with a diagnostic specificity with a lower 95% confidence bound > 92%, diagnostic sensitivities against polymerase chain reaction (PCR)–confirmed malaria for HRP2, *Pf* LDH, *Pv* LDH, and Pan LDH were 93.5%, 80.4%, 21.3%, and 55.6%, respectively. The half-life of pLDH was significantly less than that of HRP2 in thermal stability studies. Results with DBS samples collected from Peru indicate that the uncontrolled storage conditions of DBS can result in inaccurate reporting for infection with *P. falciparum* parasites with *hrp2/3* deletions. With careful consideration that minimizing the unfavorable DBS storage environment is essential for ensuring integrity of heat-labile *Plasmodium* antigens, DBS samples can be used as an alternative to liquid whole blood to detect *P. falciparum* with *hrp2/3* deletions in malaria surveillance.

**Supplementary Information:**

The online version of this article (10.1007/s12639-020-01325-2) contains supplementary material, which is available to authorized users.

## Introduction

Malaria is currently estimated to infect around 219 million individuals, resulting in 435,000 deaths each year (World Health Organization 2018). The World Health Organization aims to reduce malaria case incidence and mortality rates by at least 90% by 2030. Two human *Plasmodium* species, *P. falciparum* and *P. vivax*, cause major clinical complications, with *P. falciparum* leading to a higher mortality. Asymptomatic infections pose a problem for malaria elimination as the infected asymptomatic individuals may serve as reservoirs of parasites for continuous malaria transmission (Bousema et al. 2014; Chen et al. 2016). A significant proportion of asymptomatic infections are undetectable by microscopy and can only be detected by highly sensitive nucleic acid amplification or antigen detection assays for malaria parasite proteins (Slater et al. 2019; Jang et al. 2019; Plucinski et al. 2019; Wu et al. 2015).

*P. falciparum* histidine-rich protein 2 (HRP2) is a primary protein biomarker for malaria diagnosis. A highly sensitive rapid diagnostic test (RDT) targeting HRP2 has shown mixed results in the improvement of identifying low-level blood stage *P. falciparum* infection (Das et al. 2017; Landier et al. 2018; Das et al. 2018). All HRP2-based RDTs, which recognize a homolog of HRP2, HRP3 as well and are most widely used in Africa, can give false negative results when applied to infections with *P. falciparum* parasites with a partial or complete deletion of *hrp2* and *hrp3* (Pati et al. 2018; Kumar et al. 2013). Given these restrictions associated with HRP2, RDTs identifying *P. falciparum* with *hrp2/3* deletions and other *Plasmodium* species are needed in areas where infections with *hrp2/3* deletion mutants and other species are prevalent. Other biomarkers like *Plasmodium* lactate dehydrogenase (pLDH) and *Plasmodium* aldolase (pAldo) have been targeted in malaria RDTs (Jain et al. 2014). Especially, pLDH carrying both species-specific and Pan-specific epitopes can allow identification of these *hrp2/3* deletion mutants of *P. falciparum* and differentiate two major species and all *Plasmodium* species (Hurdayal et al. 2010).

Quantitative laboratory immunoassays for *Plasmodium* antigens have been developed on three multiplexed platforms: the simultaneous capture and sequential detection (SCSD) assay (Markwalter et al. 2016), the Luminex bead-based assay (Rogier et al. 2017; Martianez-Vendrell et al. 2020), and enzyme-linked immunosorbent assay (ELISA)-based assay using Q-Plex™ technology (Jang et al. 2019). The SCSD assay detects Pan LDH and HRP2, and the Luminex bead-based assay detects pan aldolase, Pan LDH, and HRP2. The Q-Plex^TM^ Human Malaria Array (5-Plex, Quansys Biosciences, Logan, Utah), simultaneously measures HRP2, *Pf* LDH, *Pv* LDH, Pan LDH, and CRP (Jang et al. 2020). These multiplexed assays have shown potential for screening for *P. falciparum* with *hrp2/3* deletions and the improved detection of *P. vivax* (Jang et al. 2019). Performance of the Human Malaria Array 5-Plex to date has only been demonstrated on whole blood specimens.

Whole blood samples present multiple challenges to large-scale field survey studies, including inconvenient sample collection, storage, and transport that can subsequently increase the overall cost and the accidental biohazardous risks during sample collection and transport. On the other hand, dried blood spot (DBS) samples, which are generally prepared from a finger-prick onto filter paper and subsequently dried, address these problems and are routinely collected in malaria epidemiological research studies. DBS-derived samples have been applied to assays measuring parasite nucleic acids to map the spread of drug-resistant malaria parasites and as serological markers to measure malaria transmission intensity (Pearce et al. 2003; Goodhew et al. 2014; Plucinski et al. 2018). In this study, we evaluate the use of DBS as an additional sample type for determining malaria infection via antigen detection using the Human Malaria Array 5-Plex assay. We compared assay performance with matched DBS and whole blood pellet samples from malaria-infected individuals obtained from diverse sources. We also investigated the temporal and thermal stability of each *Plasmodium* antigen and CRP on DBS stored under a range of different temperatures for up to 240 days.

## Materials and methods

### Ethics

One hundred and forty de-identified whole blood samples of asymptomatic individuals from Myanmar and Uganda were collected under informed consent and with institutional review board (IRB) approvals as described previously (Das et al. 2017). Thirty-two de-identified *P. falciparum* clinical DBS samples of symptomatic individuals were obtained at two different study sites in Peru with informed consent and with IRB approval from Universidad Peruana Cayetano Heredia (UPCH, Lima, Peru; UPCH 52707) (Gamboa et al. 2010).

### *Plasmodium* sample preparation for validation

To prepare DBS, frozen venous ethylenediaminetetraacetic acid (EDTA) blood samples collected from two study sites, Uganda and Myanmar, were thawed and used for spotting 60 µl blood per marked circle on Whatman 903 Protein saver cards (Cytiva [formerly GE Healthcare Life Sciences], Marlborough, MA) in the PATH laboratory. Care was taken to avoid touching the paper with the pipette tip by allowing the drop of blood to hang from the tip of the pipette so as to mimic DBS collection from a fingerstick blood droplet. Each card was dried overnight at room temperature (23 °C) at ambient humidity (approximately 30%) and packaged individually in Mylar bags with silica gel desiccant and humidity indicator cards (Desiccare, Inc., Las Vegas, NV). Recombinant HRP2 and CRP proteins were purchased from Microcoat Biotechnologie GmbH (Bernried am Starnberger See, Germany) and HyTest (Turku, Finland), respectively. Recombinant *Pf* LDH and *Pv* LDH proteins were purchased from MyBioSource (San Diego, CA). These recombinant malaria proteins and CRP were spiked into SeraCare™ Basematrix processed plasma (Fisher Scientific) mixed with washed red blood cells at a ratio of 6 to 4 from malaria-negative human blood purchased from PlasmaLab International (Everett, WA) to generate matched whole blood pellets and DBS samples at different protein concentrations. Malaria-negative human blood (> 10 mL) was centrifuged at 1,000 g for 10 min and washed twice with PBS. Some whole blood and DBS samples were also prepared by spiking *P. falciparum* laboratory strain W2 (BEI Resources, Manassas, VA)-infected red blood cells from in vitro culture into a malaria-negative blood pool. With the exception of DBS that were introduced into the stability test, all DBS were stored at –20 °C and < 30% relative humidity and whole blood samples were stored at –80 °C until use.

### *P. falciparum* samples with *hrp2/3* deletions from UPCH

Sample collection and storage condition for the Peru samples have been described previously for the determination of the limit of detection with the best performing HRP2-based RDTs (Gamboa et al. 2010). Finger-prick blood samples were collected on Whatman filter paper. At the time of collection, parasitemia was determined by thick smear microscopy. DBS samples were stored at –20 °C. De-identified DBS samples from two different studies conducted in 2007–2008 (Foundation for Innovative New Diagnostics [FIND] 2007, FIND 2008) and in 2014 (FIND 2014) were made available for assessment of HRP2, pLDH, and CRP using the 5-Plex. Genomic DNA isolated from DBS samples were characterized for identification of *P. falciparum* infection and genotyping of *hrp2* and *hrp3* by PCR (Gamboa et al. 2010).

### Evaluation of target analytes in DBS and whole blood

All patient DBS and whole blood samples were tested blinded to PCR results. For optimization of DBS assays, elution buffer and elution procedures involving incubation time and the addition of a shaking step were evaluated. A 6-mm diameter disc was punched out from the DBS with a sterile standard one-hole paper punch. Each dried blood disc was transferred into one well in a low-binding V-bottom 96-well plate (Thermo Scientific) with calibrator diluent (Quansys Bioscience, Logan, UT) using sterile forceps. The amount of whole blood present in one punch was estimated to be 15 μL on the basis of calculation of the applied blood volume, the measured size of the blood spread, and the assumption that the analytes are homogenous throughout the blood spot. The plate containing the blood spots was incubated overnight in 4 °C and then gently shaken at 500 rpm for 1 h at room temperature. Calibrator was reconstituted in Calibrator diluent, and 7-point dilution and blank samples were prepared for Calibration curve. The eluates were tested in two replicates on the 5-Plex using a slightly modified protocol of mixing the eluates in 4 × concentrated competitor mix to reduce the sample dilution instead of using 1 ×  competitor mix which contains the biotin-labeled CRP competitor for the competitive CRP assay. Whole blood pellet samples were tested in a single replicate on the 5-Plex after mixing with 1 ×  competitor mix (1:4). After addition of 50 µl of Calibrators and samples, the plate was incubated at room temperature with shaking at 500 rpm for 2 h. Plates were then washed with wash buffer three times. A 50-µl aliquot of detection mix was added to each well, and the plate was incubated with shaking for another hour and then washed again. For detection, a 50-µl aliquot of horseradish peroxide (HRP)-conjugated streptavidin solution was added to each well and then incubated with shaking for 30 min. After a final wash, a 50-µl aliquot of chemiluminescent substrate solution was added to each well and the chemiluminescent intensity from the array spots in each well was measured using the Q-View Imager Pro (Quansys Biosciences) at an exposure time of 300 s.

### Thermal stability experiment

Whatman 903 Protein saver cards spotted with spiked whole blood samples containing recombinant HRP2, *Pf* LDH and *Pv* LDH proteins, and CRP were dried overnight on the bench at < 30% humidity. Then, the dried cards were stored in Mylar bags containing the desiccant and held at the following temperatures: 50 °C, 30 °C, room temperature, 4 °C, and −20 °C. Samples were stored up to 240 days. At varying time points, DBS were removed from storage, and the eluates from rehydrated DBS punches were analyzed using the 5-Plex. Percentage recovery of each biomarker was calculated relative to concentration of the controls measured in reference DBS samples stored at –20 °C.

### Data analysis

Biomarker concentrations in DBS eluates were interpolated from the standard curve using a 4-parameter logistic curve-fit (Q-view imager software, Quansys Biosciences). To analyze data from the assay optimization, corrected concentrations of analytes in DBS samples were calculated from two replicate experiments by multiplication of the corresponding dilution factors (3.25 for the use of concentrated competitor and 10 for the volume of elution buffer). Results outside the limit of quantification were reported as above (> upper limit of quantification, ULOQ) or below the limit of quantification (< lower limit of quantification, LLOQ) for each analyte. The raw concentration values from the Q-view report were used for data analysis, unless indicated otherwise. Intra-assay and inter-assay variations were assessed by calculating the coefficient of variation (CV) for replicates of high, medium, and low control samples in a single plate, and 20 plates, respectively. Acceptance can be considered if CV values are less than 10% and 20% across three control samples for intra-assay and inter-assay precision, respectively (or the average of CV values for control samples is within those ranges). To assess the correlations between matched clinical whole blood and DBS samples, the Pearson correlation coefficient (*r*) was estimated. The cutoff values in DBS assays, which are equivalent to those of blood assays, was calculated from the curve’s equation obtained by means of linear regression analysis with data from matched clinical whole blood and DBS samples. The sensitivity and the specificity were calculated by the formulas (true positives)/(true positives + false negatives) and (true negatives)/(true negatives + false positives), respectively. The half-lives (t_1/2_) of biomarkers in DBS were assessed by monitoring the concentration of analytes over time with comparison to the first sample tested at each temperature. The half-life was calculated using a one phase exponential decay fit model (GraphPad Prism v.6, GraphPad Software, La Jolla, CA).

## Results

### Optimization of elution conditions for DBS samples

Procedures for extraction of malaria analytes and CRP from DBS samples were optimized by utilizing the calibrator diluent solution included in the 5-Plex kit. Variables for optimization included competitor concentration, volume of elution buffer, and a mix of elution temperature, shaking speed, and time. Increased extraction buffer volumes and more extended incubation time at room temperature led to significant improvement of recovery for all antigens throughout concentrations in five positive controls (S1, S2, S3, S4, and S5), whereas increased competitor concentration showed an effect only on the low concentration (Online Resource Figs. 1A, 1B, and 1C). Under the optimized assay procedure in which analytes are eluted from a DBS disc in 10 × elution volume at 4 °C overnight followed by incubating the eluate/disc at room temperature for 1 h in shaking, and mixed in 4 × competitor mix, the recovery of biomarkers in eluates of five control samples was examined as compared to matched blood control samples. The recovery from DBS of HRP2, *Pf* LDH, *Pv* LDH, and Pan LDH was 27%, 24%, 22%, and 27%, respectively, and 38% for CRP (Online Resource Table 1). There were excellent correlations between DBS and whole blood pellet measurements at > 0.96 of R^2^ values for all biomarkers (Online Resource Fig. 2).

### DBS-based assay repeatability

The assay variation was evaluated using positive DBS controls consisting of low, moderate, and high concentration of analytes in 20 replicates over multiple days or over a single day (Table 1). The results revealed that intra-assay average CV values for *Plasmodium* antigens met the acceptance criteria (below 10%), at each analyte concentration (Table 1). Intra-assay average CV value for CRP was 10.7%. For the inter-assay CVs the average CVs were all within the acceptance criteria (below 20%) except for CRP, which was at 25.4%. For *Plasmodium* antigens, only assays using the very low concentrations exceeded the 20% acceptance criteria (Table 1).

**Table 1:** Inter-assay and intra-assay variation of the 5-Plex testing dried blood samples. Assay variation of positive controls in dried blood (n = 20) were tested over multiple days and a single day. CVs (%) were calculated with concentration value of each analyte in DBS. Bolded are high CVs (%) above the acceptance (20% for inter-assay and 10% for intra-assay)

Analyte calculated concentration^a^		Control (n = 20 replicates)			
		Inter-assay CV (%)	Average Inter-assay CV (%)	Intra-assay CV (%)	Average Intra-assay CV (%)
HRP2	268 pg/mL	9.7	13.4	8.6	8.2
	141 pg/mL	10.3		7.9	
	12 pg/mL	**20.4**		8.2	
*Pf* LDH	2289 pg/mL	7.9	14.3	7.8	8.0
	1257 pg/mL	10.9		8.0	
	113 pg/mL	**24.0**		8.4	
*Pv* LDH	793 pg/mL	13.9	19.0	7.6	6.7
	429 pg/mL	12.7		6.7	
	37 pg/mL	**30.3**		5.8	
Pan LDH	5033 pg/mL	14.9	18.8	6.8	6.5
	2752 pg/mL	15.2		5.7	
	238 pg/mL	**26.4**		7.1	
CRP	6190 ng/mL	**22.8**	**25.4**	**10.9**	**10.7**
	2776 ng/mL	**23.0**		8.5	
	198 ng/mL	**30.6**		**12.6**	

^a^Concentration values are uncorrected for dilution of analytes in eluates

### Correlation between DBS and whole blood

**Fig. 1:**
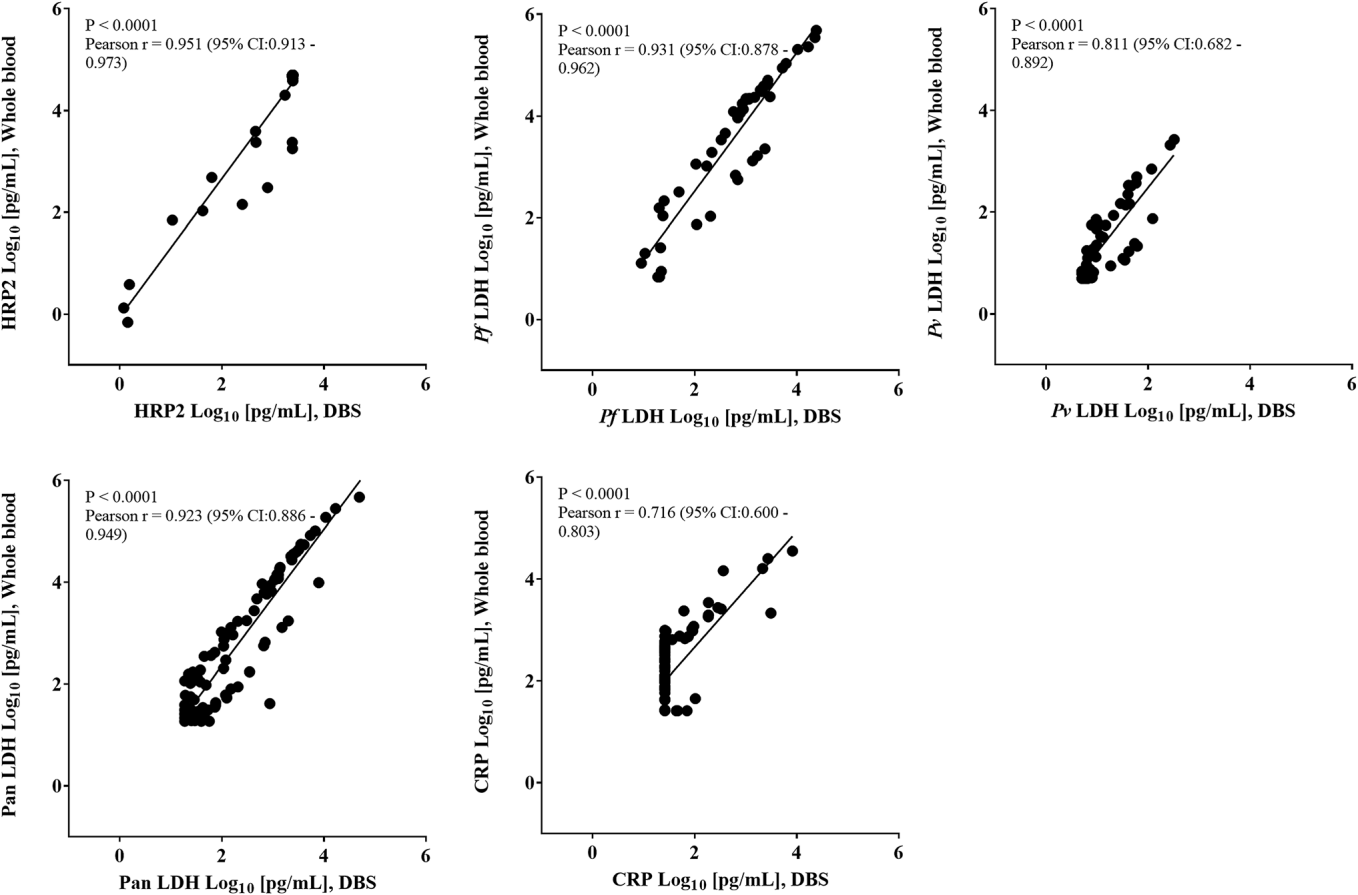
Correlation in analyte values between DBS and whole blood pellet samples. One hundred forty of the venous blood samples were from two study sites, Uganda and Myanmar, and used to prepare dried blood in the laboratory. Concentration of each analyte from DBS samples (x-axis) was plotted against concentration measured using whole blood pellet samples (y-axis). The dotted line represents the linear trend line for ideal data

To evaluate whether antigen amounts in DBS eluates are correlated to paired whole blood samples, we measured the concentration of analytes in matched DBS and blood pellets from 140 clinical samples by the 5-Plex. The biomarker concentrations in the matched DBS and whole blood pellets showed good correlation with the following Pearson correlation coefficients: HRP2 (*r* = 0.951), *Pf* LDH (*r* = 0.931), *Pv* LDH (*r* = 0.811), Pan LDH (*r* = 0.923), and CRP (*r* = 0.716) (Fig. 1). Similar to results of five positive controls in DBS, the antigen concentration values in eluates from clinical DBS samples were lower than those in the corresponding whole blood pellet samples.

### Assessment of thermal stability of biomarker in DBS over time

**Fig. 2:**
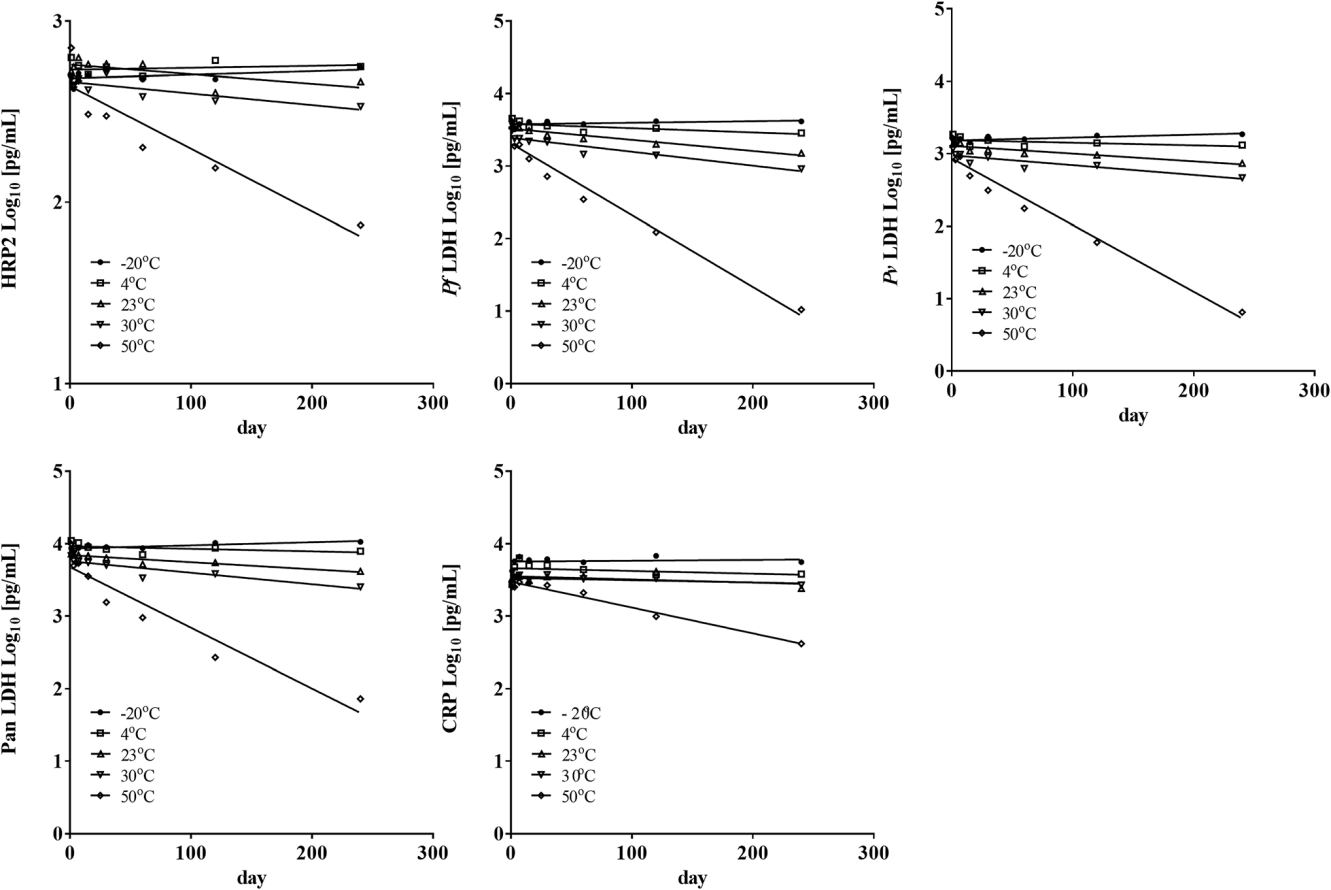
Analyte concentration recovered from DBS subjected to exposure to different temperatures over time. DBS were tested after storage at various temperature conditions for up to 240 days. The values indicate analyte concentration equivalent to the whole blood pellet sample. The dotted line represents the positive cutoff value for detection of each analyte. Dried blood samples were stored at –20 °C (close circle), 4 °C (open square), 23 °C/room temperature (open triangle), 30 °C (open inverted triangle), and 50 °C (open diamond)

To evaluate the impact of temperature on the integrity of analytes, DBS samples were kept in a sealed bag with a desiccant package at different temperatures up to 240 days. The control samples stored at −20 °C showed no change in antigen concentration over time as expected, whereas the biomarker concentrations in DBS stored at 4 °C, room temperature, 30 °C, and 50 °C decreased at increasing rates over the time (Fig. 2). The half-lives of the biomarkers in DBS were calculated from non-linear regression models based on values in DBS stored at room temperature, 30 °C, and 50 °C, and storage time (Online Resource Table 2). All *Plasmodium* antigens exhibited longer half-life at lower temperature conditions, room temperature, and 30 °C than at 50 °C. The most significant instability was observed with pLDH proteins as compared to HRP2 at each temperature condition. The CRP biomarker exhibited 88.3 days of half-life whereas *Plasmodium* antigens had a range between 12.9 and 21.6 days at 50 °C. Overall results on the decay of biomarkers after 240 days of exposure at room temperature, 30 °C, and 50 °C showed that pLDH proteins were the most temperature labile, and HRP2 and CRP proteins were the least temperature labile across the temperature ranges tested (Online Resource Table 3).

### Diagnostic performance of DBS and whole blood against PCR-confirmed infection

**Fig. 3:**
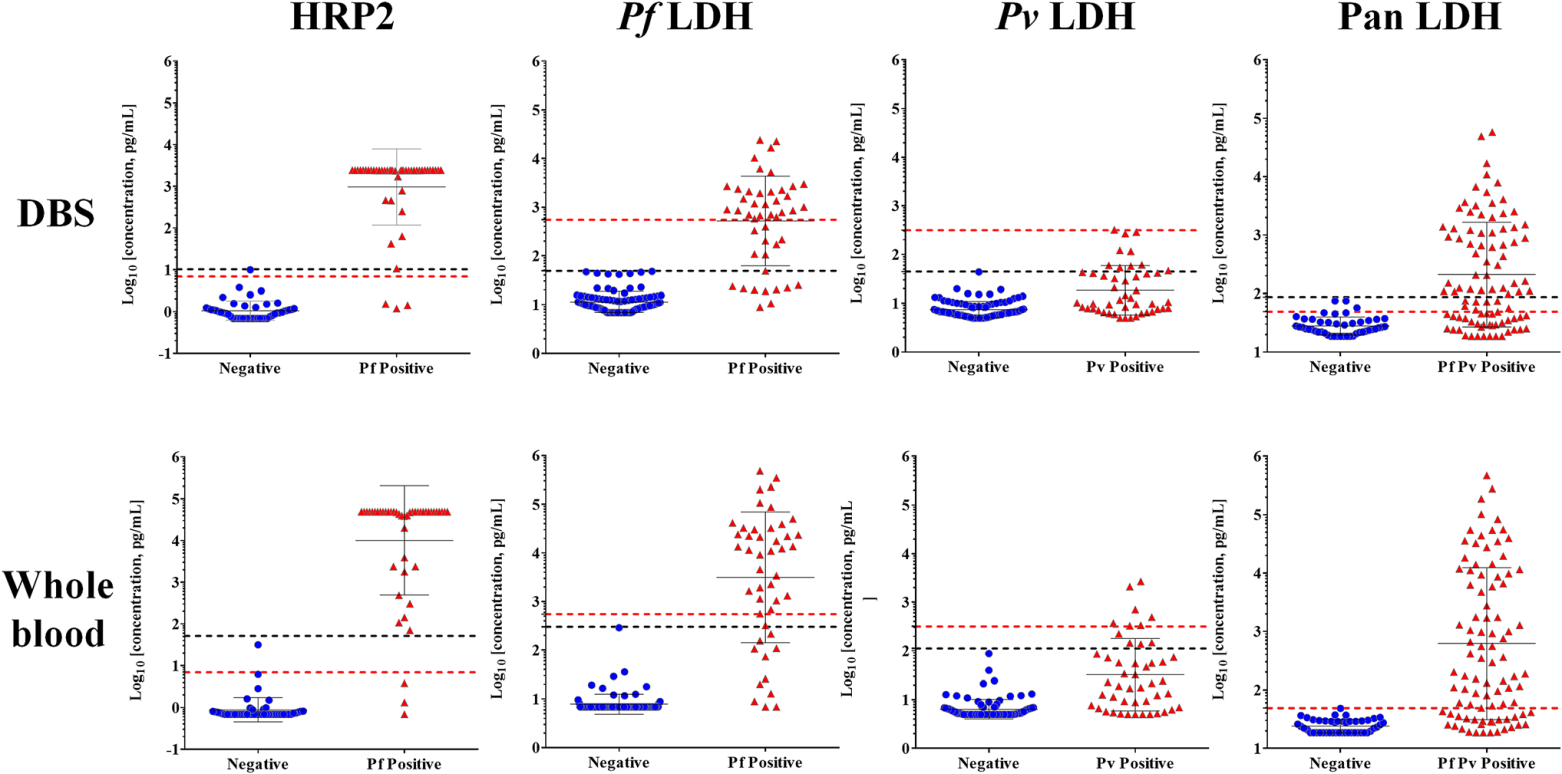
Distribution of *Plasmodium* antigens by corresponding PCR results. Scatter dot plots illustrate the log distributions of *Plasmodium* antigen concentration determined by the 5-Plex as indicated. Parasite positive and negative was determined by PCR. Black dotted line: Cutoff yielding > 99.5% specificity derived from ROC curve analysis of data from the 140 samples described here. Red dotted line: Cutoff yielding > 99.5% specificity derived from ROC curve analysis of data from 462 blood samples described previously (Jang et al. 2020). Two cutoff lines for Pan LDH are located in close proximity

In order to assess the performance of the 5-Plex assay on DBS, the 140 matched DBS and frozen whole blood specimens were analyzed against PCR for sensitivity and specificity. The distribution of biomarker concentration based on PCR-confirmed cases is shown in Fig. 3.

Receiver operating characteristic (ROC) curve analysis was conducted (Online Resource Fig. 3). The area under the curve (AUC) values for *Plasmodium* antigens are summarized in Table 2. The AUC was highest for HRP2 as a diagnostic marker for *P. falciparum* infection, equally on both DBS (0.988) and whole blood (0.982). The AUCs (0.961, 0.755, and 0.836, respectively) for DBS-based *Pf* LDH, P*v* LDH, and Pan LDH assay were lower than those (0.969, 0.844, and 0.900, respectively) for frozen blood–based assays (Table 2 and Online Resource Fig. 3). The lowest AUC was observed for *Pv* LDH on DBS and also on whole blood.

**Table 2:** Summary of area-under-the-curve (AUC) values derived from the receiver operator characteristics curve analysis (Online Resource Fig. 3) derived from the 140 matched specimens tested in this study both on DBS and whole blood pellets

	AUC (95% confidence interval)			
	HRP2	*Pf* LDH	*Pv* LDH	Pan LDH
DBS	0.988	0.961	0.755	0.836
	(0.972–1.004)	(0.924–0.997)	(0.664–0.846)	(0.772–0.901)
Whole blood	0.982	0.969	0.844	0.900
	(0.950–1.013)	(0.931–1.006)	(0.769–0.919)	(0.851–0.949)

Using the same cutoffs that were previously optimized on frozen whole blood targeting a specificity of ≥ 99.5% against PCR-confirmed infection negative frozen blood samples which were tested by the 5-Plex (Jang et al. 2020), the DBS performed with equivalency in this sample panel with whole blood with the exception of *Pv* LDH, which was significantly less sensitive (Table 3). Using the ROC curve analysis (Online Resource Fig. 3 and Online Resource Table 4), optimized DBS-specific cutoffs resulted in sensitivity and specificity estimates of the 5-Plex with DBS which were similar to those of the 5-Plex with frozen blood (Table 4 and Online Resource Table 4).

**Table 3:** Performance characteristics for each *Plasmodium* antigen assay used in the 5-Plex HRP2, *Pf* LDH for *P. falciparum, Pv* LDH for *P. vivax*, and Pan LDH for all *Plasmodium* species. The cutoff and diagnostic sensitivity/specificity for each assay was assessed in 140 of matched DBS and whole blood pellet samples. The sensitivity and specificity estimates for assays testing the DBS and frozen whole blood specimens in comparison to results of PCR as reference method are shown using cutoffs previously established for frozen whole blood specimens which were tested by the 5-Plex (Jang et al. 2020)

Target	Cutoff (pg/mL)	Source	Sensitivity (95% CI)	Specificity (95% CI)
HRP2	6.9	DBS	93.5 (82.1–98.6)	97.9 (88.7–100.0)
		Frozen blood	93.5 (82.1–98.6)	97.9 (88.7–100.0)
*Pf* LDH	553.6	DBS	63.0 (47.6–76.8)	100.0 (96.2–100.0)
		Frozen blood	76.1 (61.2–87.4)	100.0 (96.2–100.0)
*Pv* LDH	314.8	DBS	2.1 (0.1–11.3)	100.0 (96.1–100.0)
		Frozen blood	23.4 (12.3–38.0)	96.8 (90.9–99.3)
Pan LDH	49.0	DBS	63.4 (52.8–73.2)	93.6 (82.5–98.7)
		Frozen blood	72.0 (61.8–80.9)	100.0 (92.5–100.0)

**Table 4:** Performance characteristics of HRP2, *Pf* LDH, *Pv* LDH, and Pan LDH assayed on the 5-Plex from DBS. The cutoff and diagnostic sensitivity/specificity for each assay was assessed in 140 of DBS samples using receiver operating characteristic (ROC) analysis. At each cutoff value, sensitivity and specificity of the 5-Plex were calculated with their confidence interval in comparison to PCR results (Online Resource Table 4). The cutoff value to yield a specificity of 99.5% was selected

Target	Cutoff (pg/mL)	Sensitivity (95% CI)	Specificity (95% CI)
HRP2	10.4	93.5 (82.1–98.6)	100.0 (92.4–100.0)
*Pf* LDH	48.6	80.4 (66.1–90.6)	100.0 (96.2–100.0)
*Pv* LDH	45.1	21.3 (10.7–35.7)	100.0 (96.1–100.0)
Pan LDH	86.7	55.6 (45.2–66.2)	100.0 (92.5–100.0)

### Detection of *hrp2/3* deletions

The capability of the 5-Plex to identify *P. falciparum* infections that do not express HRP2 was assessed with DBS from Peru, collected over two periods of time, 2007–2008 (Study 1) and 2014 (Study 2) (Table 5). *P. falciparum* samples were confirmed for *hrp2/3* deletions by PCR. The 5-Plex failed to identify all seven *P. falciparum* infections with *hrp2/3* deletions as the *Pf* LDH assay was not able to detect *Pf* LDH in the older DBS samples from Study 1. One of these seven specimens did have detectable HRP2 above the limit of quantification (> 2464 pg/mL) by the 5-Plex, theorizing possible errors in data entry or interpretation. The repeated testing on this sample will need for confirmation. These seven samples had parasitemia in the range of 3543–10165 p/μL. On the other hand, the *Pf* LDH assay detected and measured *Pf* LDH in newer DBS samples carrying *hrp2/3*-deleted *P. falciparum* (3/4, 75%) with parasitemia in the range of 3094–13259 p/μL from Study 2. Interestingly, the 5-Plex results revealed that there were no positive HRP2 results while detecting expression of *Pf* LDH in two *P. falciparum* samples with *hrp2*^−^ *hrp3*^+^ genotype from Study 2, which had parasite densities of 5869 and 30810 p/μL, unlike to a respective sample from Study 1, which contained high HRP2 level and parasite density of 10152 p/μL.

**Table 5:** The performance of the 5-Plex to identify *P. falciparum* with *hrp2/3* deletions in DBS samples collected for two separate studies (Study 1; n = 19 and Study 2; n = 13). For determining  % Accuracy, *Pf* classification was based on positive results of either HRP2 or *Pf* LDH, and *hrp2/3* deletions were reported by detecting the absence and presence of HRP2 and *Pf* LDH, respectively

	Category	*hrp*2*/3* Deletions by PCR	5-Plex, Number of positive samples				% Accuracy of 5-Plex	
		Number of samples	HRP2	*Pf* LDH	*Pv* LDH	Pan LDH	*Pf* classification	*hrp2/3* deletions
Study 1 (2007–2008)	*Pf hrp2*^+^ *hrp3*^+^	5	5	0	0	5	100	NA^c^
	*Pf hrp2*^+^ *hrp3*^−^	6	6	0	0	5	100	NA
	*Pf hrp2*^−^ *hrp3*^+^	1	1	0	0	1	100	NA
	*Pf hrp2*^−^ *hrp3*^−^	7	2	0	0	4	28.6	0
Study 2 (2014)	*Pf hrp2*^+^ *hrp3*^+^	2	2	1	0	2	100	NA
	*Pf hrp2*^+^ *hrp3*^−^	3	3	3	0	3	100	NA
	*Pf hrp2*^−^ *hrp3*^+^	4	0^a^	2	0	4	50	NA
	*Pf hrp2*^−^ *hrp3*^−^	4	0	3^b^	0	4	75.5	75.5

^a^Predicted as *hrp2/3* deletions likely due to loss of *hrp2*, which predominantly express protein

^b^A single sample (1/4) failed to detect *Pf* LDH was reported to have 5252 p/mL of parasitemia

^c^NA: not applicable

## Discussion

In a previous study, we described the development and performance characteristics of a commercially available 5-Plex assay (Q-Plex^TM^ Human Malaria Array) for *Plasmodium* speciation using frozen whole blood samples (Jang et al. 2020). In this study we further describe the performance of the 5-Plex assay on DBS, a more readily available and convenient sample type for use in low-resource settings. The elution variables on analyte recovery, assay sensitivity and specificity, and thermal stability in DBS were assessed. Optimal elution conditions were explored using contrived DBS samples spiked with HRP2, pLDH, and CRP dried onto Whatman 903 cards (Online Resource Fig. 1). The results revealed that a longer incubation with shaking and increasing larger elution volumes resulted in significant recoveries for the analytes. Additionally, the use of a concentrated competitor mix helped to lower the limit of detection. Using the optimal elution procedure, the recovery of analytes from DBS samples were not equivalent to whole blood pellet samples, indicating the limitation of efficiency of analyte elution from DBS (Online Resource Table 1). Further research efforts are needed to increase the yield allowing efficient recovery from DBS and facilitating the utility of DBS matrices for antigen measurement.

The inter-assay and intra-assay variation experiments on DBS showed acceptable reproducibility for *Plasmodium* antigens (Table 1) except at the very lower level of analyte where the CV exceeded 20%. The variation observed with inter-assay and intra-assay for CRP were exceeded 20% and 10% of CV, respectively. The source of this variation in CRP should be further investigated, taking into consideration that the CRP is measured by competitive ELISA in the 5-Plex assay.

A stability study on *Plasmodium* antigens in DBS demonstrated that the antigens are better preserved in dried blood samples packaged with desiccants and stored at lower temperature, preferably at −20 °C (Fig. 2, and Online Resource Tables 2 and 3). A similar trend has been seen with different analytes in other studies when stored on DBS (Brindle et al. 2019; Gibson et al. 2017). Overall LDH had a consistently shorter half-life than HRP2 at all temperatures including 4 °C, with the exception of −20 °C. Markwalter et al. (2018) previously reported the loss of both pLDH and HRP2 in DBS by 70% after 8 days of storage at room temperature. It would be interesting to investigate if the use of native versus recombinant proteins is a reason for this disagreement. The higher temperature lability characteristics of pLDH has large implications on the use of DBS for screening for infections carrying *P. falciparum* with *hrp2/3* deletions, which relies solely on pLDH detection. Our results show that DBS stored for long periods of time may not be reliable for pLDH-based detection of malaria infection be it *hrp2/3* deletions or non-falciparum infections. Furthermore, DBS should be stored with desiccant at a minimum of −20 °C for extended storage. The poor stability of pLDH imposes the requirement for maintaining good storage conditions and custody for the DBS to ensure reliability of antigen detection assays downstream. While this is also a requirement for the whole blood pellets, typically storage conditions for DBS are not monitored as closely. However, the biomakers are preserved for short period of time if DBS are properly prepared and so may be collected and shipped to a facility for long term cold storage without significant decay.

Clinical samples showed a high correlation between concentration of antigens in matched whole blood and DBS (Fig. 1). For the AUC values obtained from the ROC curve analyses conducted on the HRP2, *Pf* LDH, *Pv* LDH, and Pan LDH data, those for DBS and whole blood-based HRP2 assays were the highest, indicating that HRP2 assay is very accurate (Table 2). Using PCR results as reference, the sensitivity and specificity of the 5-Plex in diagnosing malaria infection were calculated. Cutoff values previously established for frozen whole blood specimens (Jang et al. 2020) led to the similar sensitivity and specificity estimates for HRP2 assay in DBS and whole blood, but lower sensitivity and specificity estimates for pLDH assays in DBS (Table 3). These results indicate that there was more difference between DBS and whole blood with regards to the reactivity of pLDH. However, the ROC curve analysis using data from140 DBS samples recommended different cutoff values, but similar sensitivity and specificity estimates especially for HRP2, *Pf* LDH and Pan LDH assays. Significant limitations of this study could be the relatively small number of matched DBS and frozen blood specimens, and the smaller concentration range for each analyte resulted from including only asymptomatic malaria cases from two geographic regions. Consequently, the cutoffs for the 5-Plex using DBS-derived samples tested here may not be optimal and should be further validated with more specimens and from a broader geography.

In addition, our data from a very small sample size suggests that reliable malaria speciation and *hrp2/3* deletions screening can be performed on DBS even after at least 4 years storage as long as the DBS have been stored appropriately at –20 °C (Table 5). Most of anti-HRP2 antibodies can react to HRP3 which has a sequence homology in the histidine-rich repeat region of HRP2. However, no detectable amount of HRP2 protein found in *P. falciparum* with *hrp2*^−^ *hrp3*^+^ genotype from Study 2 suggests that the cross-reactivity to HRP3 did not increase the detectability of HRP2 assay due to the lower abundance of HRP3 (Baker et al. 2011).

We previously reported that the increased levels of CRP, an acute phase inflammatory protein are found in febrile malaria cases (Jang et al. 2020). CRP level can predict the risk of complications in febrile malaria infection. Examining the distribution of CRP concentration based on PCR results showed that there was no significant difference between healthy individuals and asymptomatic malaria-positive individuals when DBS and whole blood samples were tested (Online resources Fig. 4).

Although these results are preliminary and need more data from a larger sample set to be conclusive, the present study demonstrates that the 5-Plex can utilize DBS as a sample type, increasing the utility of the 5-Plex as a simple and sensitive test to inform on the dynamics of malarial species in low prevalence settings and with the emergence of *P. falciparum* with *hrp2/3* deletions.

## Supplementary Information

Below is the link to the electronic supplementary material.

Supplementary file1 (DOCX 476 kb)

## Data Availability

Not Applicable
