# Supplementary material for: Assessment of Plasmodium antigens and CRP in dried blood spots with multiplex malaria array
Source: J Parasit Dis. 2021 Jan 3;45(2):479–89. doi: 10.1007/s12639-020-01325-2 (PMC8254675; doi:10.1007/s12639-020-01325-2)
Supplement: Supplementary file 1 — Supplementary file1 (DOCX 476 kb) [file 12639_2020_1325_MOESM1_ESM.docx]

**Journal of Parasitic Diseases**

**Assessment of *Plasmodium* antigens and CRP in dried blood spots with multiplex malaria array**

Ihn Kyung Jang,^*^ Sara Aranda, Rebecca Barney, Andrew Rashid, Muhammad Helwany, John C. Rek, Emmanuel Arinaitwe, Harriet Adrama, Maxwell Murphy, Mallika Imwong, Stephane Proux, Warat Haohankhunnatham, Xavier C. Ding, François Nosten, Bryan Greenhouse, Dionicia Gamboa, and Gonzalo J. Domingo

*** Corresponding author:** Ihn Kyung Jang, Diagnostics Program, PATH, Seattle, Washington, USA; [ikjang@path.org](mailto:ikjang@path.org)

**A**

**B**

**C**

**Online Resource Fig 1** Optimization of DBS-based 5-Plex. Five positive controls prepared in the successive dilutions of recombinant proteins were tested to evaluate three variables for assay optimization: A) competitor, B) elution buffer volume, and C) overnight incubation at 4^o^C followed by additional shaking at room temperature. Experimental results were analyzed by a two-way analysis of variance (ANOVA). Corrected concentration values accounting for dilution of eluates were used for further analysis. When overall significance was achieved, the Holm-Sidak post-hoc test (for ANOVAs) was used to make all possible comparisons.

**Online Resource Table 1** Average recovery percentage of analytes in DBS eluates in comparison to matched whole blood pellets. Antigen concentration in five positive controls prepared by the successive dilutions of recombinant proteins (S1, S2, S3, S4, S5) were determined by the 5-Plex assay. Corrected concentration values accounting for dilution of eluates were used for further analysis.

|  | **HRP2 (n = 5)** | ***Pf* LDH (n = 5)** | ***Pv* LDH (n = 5)** | **Pan LDH (n = 5)** | **CRP**  **(n = 5)** |
| --- | --- | --- | --- | --- | --- |
| **Average %**  **recovery compared to whole blood (± SD)** | 26.9 ± 11.3 | 23.9 ± 9.8 | 21.6 ± 10.1 | 27.1 ± 11.7 | 38.0 ± 16.7 |

**
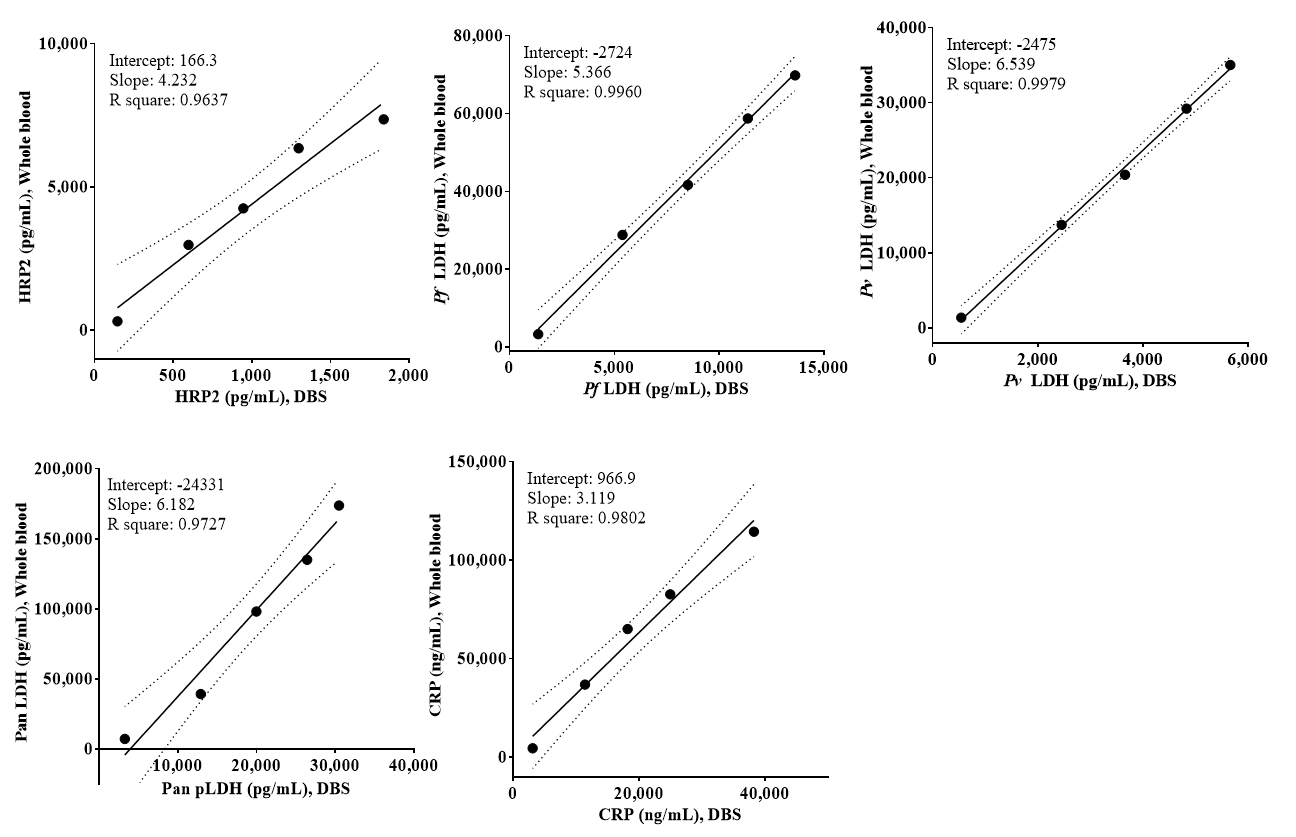
**

**Online Resource Fig 2** Comparison between matched DBS and whole blood pellet samples. Antigen concentration in five positive controls prepared by the successive dilutions of recombinant proteins (S1, S2, S3, S4, S5) were determined by the 5-Plex assay. Corrected concentration values accounting for dilution of eluates were used for further analysis. The linear regression analysis was performed for regression line of best fit for each assay. Solid line, regression line; dotted area, 95% confidence interval.

**Online Resource Table 2** Half-lives in days for *Plasmodium* antigens and CRP in DBS. The times for total antigens to fall to 50% of initial concentration in DBS were extrapolated using exponential decay fit model. Analyte concentrations were determined by the 5-Plex after storing DBS at the different storage temperature (room temperature, 30^o^C, and 50^o^C) and period (day 1–240). NA: Apparent instability of CRP was not observed at room temperature and 30^o^C within the given time period.

| **Target** |  | **Half-life (in days) calculated using exponential decay fit** | | |
| --- | --- | --- | --- | --- |
|  | **RT^a^** | | **30oC** | **50oC** |
| HRP2 | 88.0 (R^2^= 0.55) | | 84.9 (R^2^= 0.69) | 21.6 (R^2^= 0.88) |
| *Pf* LDH | 51.4 (R^2^= 0.98) | | 42.6 (R^2^= 0.92) | 12.9 (R^2^= 0.90) |
| *Pv* LDH | 51.9 (R^2^= 0.89) | | 35.7 (R^2^= 0.80) | 13.9 (R^2^= 0.92) |
| Pan LDH | 64.0 (R^2^= 0.93) | | 37.3 (R^2^= 0.90) | 13.2 (R^2^= 0.93) |
| CRP | NA**^b^** | | NA | 88.3 (R^2^= 0.96) |

**^a^ RT: room temperature**

**^b^ NA: not applicable**

**Online Resource Table 3** Percentage loss of analytes after exposure of DBS to different temperatures for 1 day and 240 days. Analyte concentrations in DBS samples at the given day were compared to those at day 0. The values superior to the acceptable inter-assay CV (%) (> 20% for antigen) are shown in bold.

| **Temperature** | **% loss of analyte after exposure to different temperature for 1 day and 240 days** | | | | | | | | | |
| --- | --- | --- | --- | --- | --- | --- | --- | --- | --- | --- |
|  | **HRP2** | | ***Pf* LDH** | | ***Pv* LDH** | | **Pan LDH** | | **CRP** | |
|  | **1** | **240** | **1** | **240** | **1** | **240** | **1** | **240** | **1** | **240** |
| –20^o^C | –14.6 | –5.7 | –13.5 | –8.1 | –10.6 | 4.7 | –11.5 | 8.6 | 28.5 | **70.4** |
| 4^o^C | 6.9 | –4.8 | 1.1 | **–36.4** | –4.2 | **–25.8** | 12.6 | –19.5 | –16.1 | 16.2 |
| RT | –10.3 | **–21.2** | –19.8 | **–66.7** | **–23.5** | **–58.5** | **–26.1** | **–57.6** | 1.2 | **–26.4** |
| 30^o^C | –18.2 | **–42.5** | **–30.6** | **–79.7** | **–33.8** | **–73.7** | **–30.2** | **–74.2** | –18.2 | –18.1 |
| 50^o^C | **–20.9** | **–87.3** | –8.6 | **–99.8** | –8.4 | **–99.6** | 1.1 | **–99.3** | **–21.9** | **–87.3** |

**
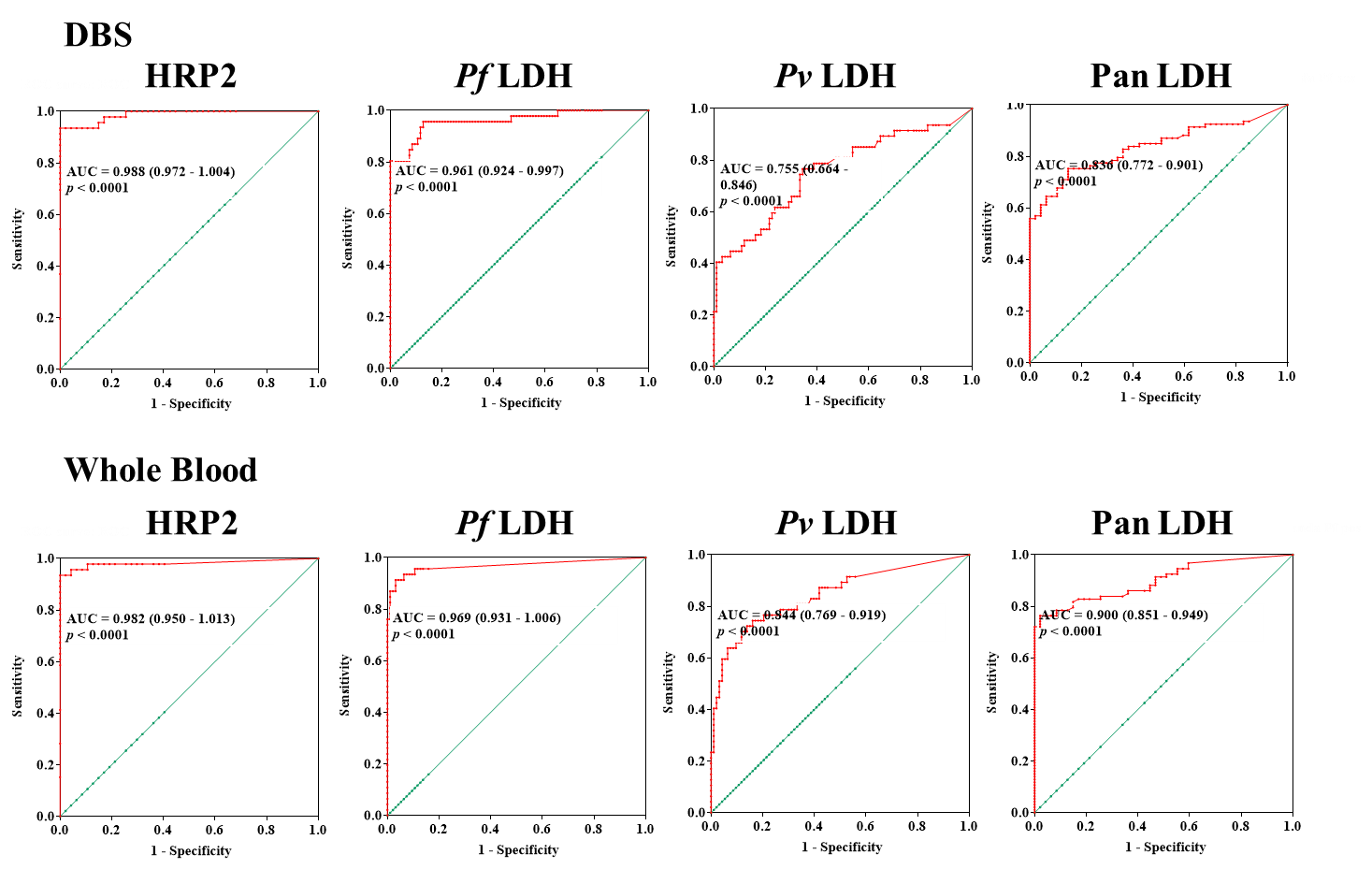
**

**Online Resource Fig 3** Receiver operating characteristic (ROC) curves demonstrating the true positive rate (sensitivity) as function of false positive rate (1– specificity) for detecting HRP2, *Pf* LDH, P*v* LDH, and pan LDH in matched DBS and whole blood pellet samples (n = 140). The red line shows the mean area under the curve (AUC) plot with the AUC value and the 95% confidence intervals in parentheses.

**Online Resource Table 4** Sensitivity and specificity resulting from a particular cutoff value. The table displays only the representative data points that are higher than 0.85 of specificity.

**DBS**

| Cutoff (pg/mL) | Sensitivity | 95% CI | Specificity | 95% CI |
| --- | --- | --- | --- | --- |
| HRP2 |  |  |  |  |
| > 3.445 | 0.9348 | 0.8210 to 0.9863 | 0.9574 | 0.8546 to 0.9948 |
| > 6.885 | 0.9348 | 0.8210 to 0.9863 | 0.9787 | 0.8871 to 0.9995 |
| > 10.35 | 0.9348 | 0.8210 to 0.9863 | 1.000 | 0.9245 to 1.000 |
| *Pf* LDH |  |  |  |  |
| > 15.53 | 0.9565 | 0.8516 to 0.9947 | 0.8511 | 0.7628 to 0.9161 |
| > 21.72 | 0.8696 | 0.7374 to 0.9506 | 0.9043 | 0.8260 to 0.9553 |
| > 43.67 | 0.8043 | 0.6609 to 0.9064 | 0.9574 | 0.8946 to 0.9883 |
| > 46.50 | 0.8043 | 0.6609 to 0.9064 | 0.9787 | 0.9252 to 0.9974 |
| > 47.49 | 0.8043 | 0.6609 to 0.9064 | 0.9894 | 0.9421 to 0.9997 |
| > 48.59 | 0.8043 | 0.6609 to 0.9064 | 1.000 | 0.9615 to 1.000 |
| *Pv*LDH |  |  |  |  |
| > 11.16 | 0.4894 | 0.3408 to 0.6394 | 0.8602 | 0.7728 to 0.9234 |
| > 13.18 | 0.4468 | 0.3017 to 0.5988 | 0.9032 | 0.8242 to 0.9548 |
| > 15.59 | 0.4255 | 0.2826 to 0.5782 | 0.9570 | 0.8935 to 0.9882 |
| > 19.43 | 0.4043 | 0.2637 to 0.5573 | 0.9785 | 0.9245 to 0.9974 |
| > 20.36 | 0.4043 | 0.2637 to 0.5573 | 0.9892 | 0.9415 to 0.9997 |
| > 45.14 | 0.2128 | 0.1070 to 0.3566 | 1.000 | 0.9611 to 1.000 |
| Pan LDH |  |  |  |  |
| > 37.60 | 0.7527 | 0.6524 to 0.8363 | 0.8511 | 0.7169 to 0.9380 |
| > 46.96 | 0.6452 | 0.5391 to 0.7417 | 0.9149 | 0.7962 to 0.9763 |
| > 59.19 | 0.6129 | 0.5062 to 0.7122 | 0.9574 | 0.8546 to 0.9948 |
| > 75.13 | 0.5699 | 0.4631 to 0.6722 | 0.9787 | 0.8871 to 0.9995 |
| > 86.74 | 0.5591 | 0.4524 to 0.6620 | 1.000 | 0.9245 to 1.000 |

**Whole Blood**

| Cutoff (pg/mL) | Sensitivity | 95% CI | Specificity | 95% CI |
| --- | --- | --- | --- | --- |
| HRP2 |  |  |  |  |
| > 0.9450 | 0.9783 | 0.8847 to 0.9994 | 0.8511 | 0.7169 to 0.9380 |
| > 1.560 | 0.9565 | 0.8516 to 0.9947 | 0.9149 | 0.7962 to 0.9763 |
| > 3.325 | 0.9565 | 0.8516 to 0.9947 | 0.9574 | 0.8546 to 0.9948 |
| > 18.87 | 0.9348 | 0.8210 to 0.9863 | 0.9787 | 0.8871 to 0.9995 |
| > 50.85 | 0.9348 | 0.8210 to 0.9863 | 1.000 | 0.9245 to 1.000 |
| *Pf* LDH |  |  |  |  |
| > 7.600 | 0.9565 | 0.8516 to 0.9947 | 0.8617 | 0.7751 to 0.9243 |
| > 10.68 | 0.9348 | 0.8210 to 0.9863 | 0.9043 | 0.8260 to 0.9553 |
| > 18.63 | 0.9130 | 0.7921 to 0.9758 | 0.9574 | 0.8946 to 0.9883 |
| > 32.34 | 0.8696 | 0.7374 to 0.9506 | 0.9787 | 0.9252 to 0.9974 |
| > 54.72 | 0.8696 | 0.7374 to 0.9506 | 0.9894 | 0.9421 to 0.9997 |
| > 305.4 | 0.7609 | 0.6123 to 0.8741 | 1.000 | 0.9615 to 1.000 |
| *Pv* LDH |  |  |  |  |
| > 8.505 | 0.7234 | 0.5736 to 0.8438 | 0.8602 | 0.7728 to 0.9234 |
| > 11.21 | 0.6596 | 0.5069 to 0.7914 | 0.9032 | 0.8242 to 0.9548 |
| > 13.26 | 0.5957 | 0.4427 to 0.7363 | 0.9570 | 0.8935 to 0.9882 |
| > 37.04 | 0.4043 | 0.2637 to 0.5573 | 0.9785 | 0.9245 to 0.9974 |
| > 43.39 | 0.4043 | 0.2637 to 0.5573 | 0.9892 | 0.9415 to 0.9997 |
| > 112.9 | 0.2340 | 0.1230 to 0.3803 | 1.000 | 0.9611 to 1.000 |
| Pan LDH |  |  |  |  |
| > 31.08 | 0.8172 | 0.7235 to 0.8898 | 0.8511 | 0.7169 to 0.9380 |
| > 34.29 | 0.7849 | 0.6876 to 0.8634 | 0.9149 | 0.7962 to 0.9763 |
| > 37.31 | 0.7634 | 0.6640 to 0.8454 | 0.9574 | 0.8546 to 0.9948 |
| > 37.98 | 0.7634 | 0.6640 to 0.8454 | 0.9787 | 0.8871 to 0.9995 |
| > 48.98 | 0.7204 | 0.6178 to 0.8086 | 1.000 | 0.9245 to 1.000 |


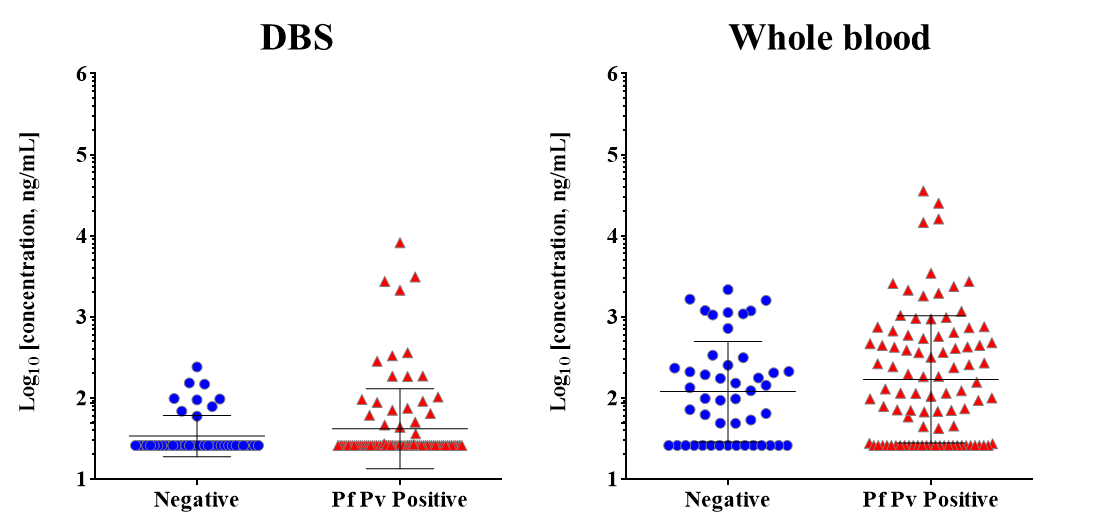


**Online Resource Fig 4** Distribution of CRP by corresponding PCR results. Scatter dot plots illustrate the log distributions of CRP concentration in DBS and whole blood samples determined by the 5-Plex as indicated.
